# The predictive ability of ABSI compared to BMI for mortality and frailty among older adults

**DOI:** 10.3389/fnut.2024.1305330

**Published:** 2024-04-12

**Authors:** Itamar Shafran, Nir Y. Krakauer, Jesse C. Krakauer, Abigail Goshen, Yariv Gerber

**Affiliations:** ^1^Department of Epidemiology and Preventive Medicine, School of Public Health, Faculty of Medicine, Tel Aviv University, Tel Aviv, Israel; ^2^Department of Civil Engineering, City College of New York, New York, NY, United States; ^3^Associated Physicians/Endocrinology, Berkley, MI, United States

**Keywords:** ABSI, BMI, mortality, frailty, healthy aging

## Abstract

**Introduction:**

To study the utility of A Body Shape Index (ABSI) alongside body mass index (BMI) to predict mortality and frailty in an aging community population.

**Materials and methods:**

Participants (*n* = 1,580) were drawn from the first Israeli national health and nutrition survey of older adults (“Mabat Zahav”) conducted from 2005 to 2006, constituting adults aged ≥65 years. Socio-demographic, clinical, behavioral, and psychosocial data were collected. Baseline weight, height, and waist circumference (WC) were measured and expressed as the allometric indices BMI (kg/m^2^) and ABSI, a BMI-independent measure of abdominal obesity [WC/(BMI^2/3^*m^1/2^)]. Mortality follow-up lasted through 2019. Frailty was assessed in 2017–2019 by the Fried Biological Phenotype in a sub-cohort of 554 survivors. Cox and logistic regression models assessed associations of BMI and ABSI with mortality and frailty.

**Results:**

At baseline, mean [SD] age was 74.5 [6.1] years, and 52.4% were women. The correlation between BMI and WC Z scores was 0.71, reduced to −0.11 for BMI and ABSI. Over a median follow-up of 13 years, 757 deaths occurred. The multivariable-adjusted hazard ratios (95% CIs) for mortality per standard deviation increase in BMI and ABSI were 1.07 (0.99;1.17) and 1.13 (1.05;1.21), respectively. Among participants assessed for frailty, 77 (14%) met the frailty criteria. After multivariable adjustment, the odds ratios (95% CIs) for frailty were 0.83 (0.69–1.01) for BMI and 1.55 (1.34–1.79) for ABSI.

**Discussion:**

In a nationwide cohort of older adults, ABSI was independently associated with mortality risk. Furthermore, ABSI, but not BMI, was a strong predictor of frailty.

## Introduction

With increasing longevity, the importance of healthy aging or “health span” is being recognized as equally or often more important than medical events and morbidities. Healthy aging determines many aspects of quality of life and the need for long-term care in a skilled nursing facility (1, 2).

The prevalence of frailty in community older adults aged 65 and above is reported to be around 11–12% (3, 4), and it is one of the greatest challenges in this growing population (5). The assessment of frailty is often based on Fried’s Biological Phenotype (6). With an insidious onset and slow progression, the identification of individuals at increased risk has become an important focus in geriatrics to facilitate the prevention of frailty and its dire consequences.

Abdominal obesity is visually identified as anterior bulging. Quantitatively, the definition of abdominal obesity is most often based on waist circumference (WC), with sex-and ethnic-specific thresholds identified. However, height, weight, and WC are highly correlated. Indeed, the correlation between BMI and WC is approximately 0.7–0.9, which means that above a certain BMI, the criteria for “abdominal obesity” by WC will be met. For example, in 99.8% of individuals with BMI >35, WC was above the threshold for abdominal obesity (7). In 2012, A Body Shape Index (ABSI) was defined to approximate WC normalized by the expected value of WC from weight and height (8), analogous to BMI, which is defined to normalize weight for height. The correlation of ABSI with BMI is thereby minimized, to provide a BMI-independent measure of the abdominal bulge (body shape as opposed to size). ABSI has been validated as a robust predictor of mortality risk in diverse observational studies (9–14). Relative to a model based on lipids, ABSI was found to better predict cardiovascular disease (CVD) in men (10). Relative to the metabolic syndrome (MS), ABSI was a better predictor of mortality, which could be further improved by adding the non-WC components of the MS score (15). Recently, ABSI has been proposed as a substitute for WC in the definition of MS (16, 17). Risk attributable to ABSI has been shown to add approximately multiplicatively to risk attributable to BMI, with the concept of anthropometric risk index (ARI) devised to capture the summed risk, for example for mortality, from multiple independent anthropometric indices (18).

The present study is a continuation of research into healthy aging, specifically, the absence of frailty, with data from “Mabat Zahav” (the Israeli National Health and Nutrition Survey of Older Adults) (19, 20). We sought to assess the applicability of ABSI to the geriatric population sample and the ability of ABSI alongside BMI to predict mortality and frailty over a decade of observation.

## Materials and methods

### Study design

**Figure 1:**
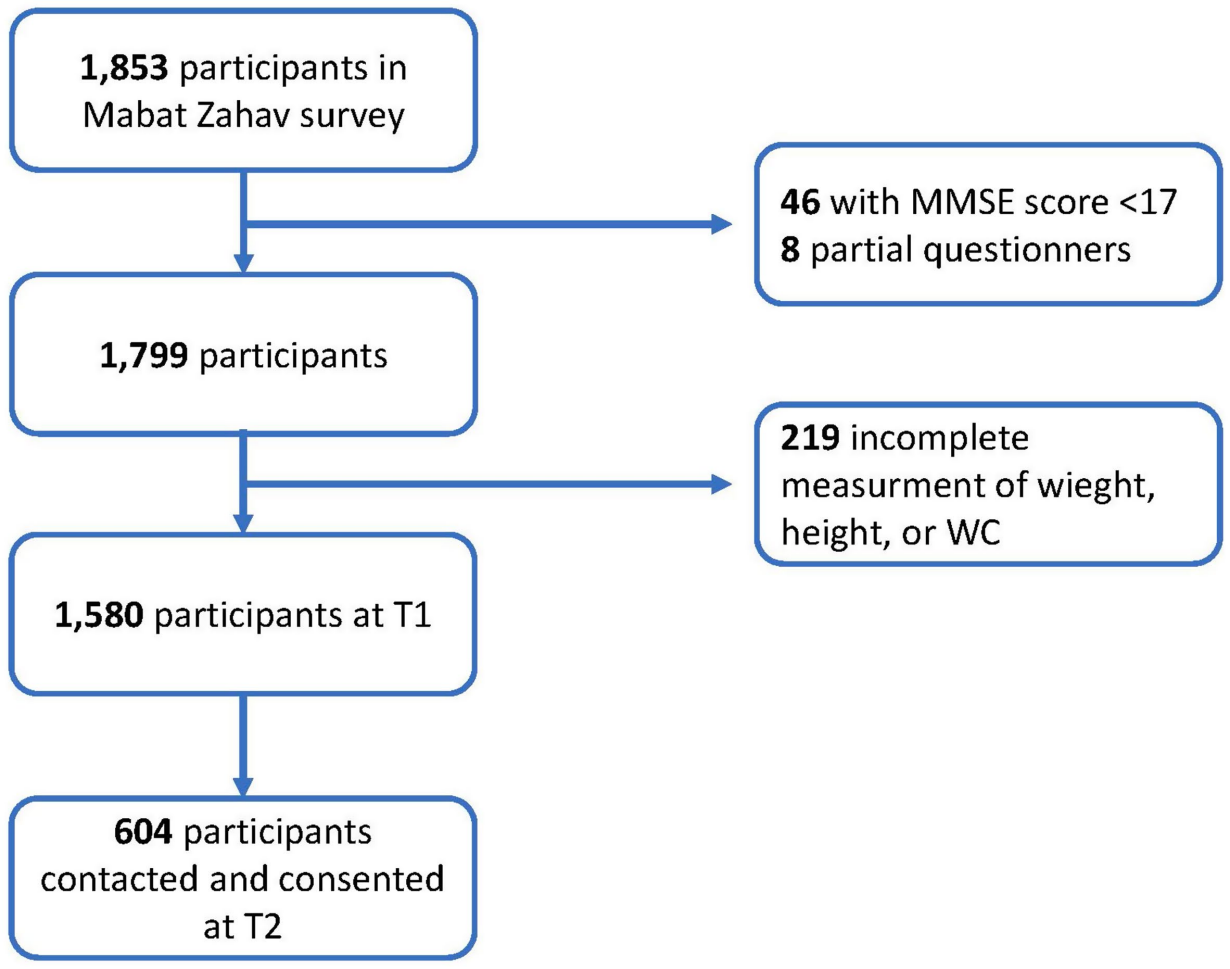
Flowchart of the study sample. MMSE, Mini-Mental Status Examination; T1, time 1 (July 2005 to December 2006); and T2, time 2 (May 2017 to June 2019).

In our study we examined the predictive role of a new anthropometric measurement ABSI in the development of frailty and mortality incidence, using a cohort study design. The study was based on the First Israeli National Health and Nutrition Survey of Older Adults (“Mabat Zahav”) that was conducted from July 2005 to December 2006 (time 1 [T1]). The survey population consisted of 1,853 community-dwelling participants aged 65 and above; data collection included information regarding health and nutrition status, functional and cognitive function, and anthropometric measurements. The survey was carried out by the Israel Center for Disease Control (ICDC) and the Nutrition Department of the Israel Ministry of Health (MOH). After excluding participants with low Mini-Mental State Exam (MMSE) (21) scores and incomplete questionnaires, the final study population at T1 consisted of 1,799 participants, of whom 1,580 participants had complete weight, height, and WC data. From May 2017 to June 2019, after a median duration of 12.5 (IQR: 11.8–13.1) years, survivors of the original survey were contacted, and an extensive face-to-face interview and functional assessment were conducted among 604 participants [time 2 (T2)] (Figure 1).

The “Mabat Zahav” population was a random sample of community-dwelling Israeli citizens aged ≥65 years (1,536 Jews and 316 Arabs). Exclusion criteria included significant cognitive reduction (MMSE <17), long-term hospitalization at the time of the study, and severe dementia. Participants were personally interviewed at their homes or nursing institutes, using a structured questionnaire in the participant’s language.

The sampling frame included adults aged 65 and above, who were members of one of the two major Health Maintenance Organizations (HMOs) in Israel: Clalit Health Services and Maccabi Health Services, which represented 86.3% of all older adults in Israel at the time of the survey. The Arab population was oversampled, due to the small percentage of elderly in this population (6.3% above the age of 65), to ensure an adequate sample size for statistical analyses. The overall sample size target was 1,800 participants (1,500 Jews and 300 Arabs). At T2, 486 Jewish and 115 Arab participants were contacted and consented to participate in the follow-up interview (19, 20).

### Allometric measurements

Height, body weight, and WC were measured at T1. Height and weight were used to calculate BMI (kg/m^2^). Complete data were available for 1,580 participants. ABSI was calculated as the ratio of measured WC (m) to the expected WC calculated as a function of weight (kg) and height (m) (8), as follows:

$$\mathrm{ABSI}=\left(WC\dot{{\mathrm{weight}}^{\frac{-2}{3}}}{\mathrm{height}}^{\frac{5}{6}}\right)=WC/\left({BMI}^{\frac{2}{3}}*{\mathrm{height}}^{\frac{1}{2}}\right)$$

ARI was calculated using the BMI and ABSI, representing a combined estimated risk for mortality from both indices (18):

$$ARI=\ln\left({\mathrm{OR}}_{BMI}\right)+\ln\left({\mathrm{OR}}_{\mathrm{ABSI}}\right)$$

Where ln is natural logarithm and OR is the odds ratio for mortality as a function of either BMI or ABSI Z score as estimated based on a United States population sample (18). OR_ABSI_ increased quasi-exponentially with ABSI Z score, while OR_BMI_ was elevated for both very low and very high BMI levels.

Finally, we converted the values of height, BMI, ABSI, and ARI to standardized Z scores for further analysis.

### Frailty score

Frailty was assessed using Fried’s Biological Phenotype during the follow-up interview at T2 only (20). According to Fried’s Biological Phenotype, frailty is determined by the presence of three or more of the following: (1) Shrinking: unintentional weight loss of more than 4.5 kg, or more than 5% of body weight in the previous year; (2) Weakness: grip strength in the lowest 20%, adjusted for sex and BMI; (3) Poor endurance and energy: indicated by self-report of exhaustion; (4) Slowness: the slowest 20% of participants in a 5-meter walk, adjusted for sex and standing height; (5) Low physical activity level: the lowest quintile of a weighted score of kilocalories expended per week, based on the physical activity scale for the elderly questionnaire, each sex separately. The participants were then categorized into frail or non-frail groups.

### Mortality

Original participants were matched by their national identification numbers to the National Death Registry (last updated in June 2019).

### Covariates

Covariates used in the analysis included age, sex, ethnicity (Jewish vs. Arab), smoking status (self-reported), MMSE score, comorbidities, and neighborhood socioeconomic status (SES). Comorbidities were self-reported and included heart attack, heart failure, other cardiac disease, stroke / cerebrovascular accident (CVA), cataract, glaucoma, chronic renal failure, cancer, Alzheimer’s disease, Parkinson’s disease, asthma, other lung disease, diabetes, osteoporosis, dyslipidemia, and hypertension. SES was estimated by the Israel Central Bureau of Statistics on a 20-point scale based on the 1995 national census (22). We grouped the SES variable into tertiles and labeled them accordingly as low, mid, and high SES groups, corresponding with the scores of 0–9, 10–13, and 14–20, respectively. All covariates were assessed at the beginning of the study (T1) as part of the “Mabat Zahav” survey.

### Statistical analysis

Analysis was performed using R version 4.0.4 (The R Foundation for Statistical Computing). Survival analysis was conducted using Cox regression models, where participants who survived until the end of follow-up (June 2019) were right censored. The proportional hazards assumption was tested using the Schoenfeld residuals, with no violations found in any of the models. To better visualize the association of each anthropometric measurement and the hazard ratio (HR) for mortality, we plotted log HR against the Z scores of the anthropometric measurement, thus allowing us to demonstrate non-linear associations between the continuous anthropometric variables and mortality. The reference level for each HR curve was the mean Z score for that anthropometric measure. Logistic regression models were used to assess the relation between BMI, ABSI, and ARI with frailty. Because many participants were not able to attend the T2 interview, we used inverse probability weighting (IPW) to minimize selection bias due to attrition. The probability of participating in the follow-up interview at T2 was calculated using a binominal logistic regression model. The weights were calculated as the reciprocal value of the probability (23).

We used three hierarchical models in the analysis, where every model is based on the previous one with further adjustment for additional covariates. Accordingly, the models were adjusted for age, sex, ethnicity, SES, and comorbidities. Additional covariates were adjusted for in sensitivity analyses. The potential confounders were selected as they represent risk factors for mortality and frailty. Participants with missing frailty information or anthropometric measurement data were excluded from the analysis. We used a missing indicator for covariates with missing values exceeding 1%.

## Results

The analysis included 1,580 participants, of whom 52.4% were female, and the mean [SD] age was 74.5 [6.1] years. Baseline characteristics across tertiles of each Z score of the anthropometric measures are shown in Table 1. There was no significant difference in mean age between the different levels of each anthropometric measurement. Significant differences were noted for sex (higher percentage of females with higher BMI and lower ABSI and ARI), Arab ethnicity (higher proportion with higher BMI, ABSI, and ARI), and mean number of comorbidities (higher with higher BMI, ABSI, and ARI).

**Table 1:** Baseline characteristics across anthropometric measures tertiles.

	BMI			*p* value	ABSI			*p* value	ARI			*p* value	Height			*p* value
Tertiles*	Low (16.02,26.84)	Mid (26.84,30.76)	High (30.78,54.31)		Low (0.062,0.079)	Mid (0.079,0.085)	High (0.085,0.100)		Low (−0.37, −0.15)	Mid (−0.15, 0.01)	High (0.01, 0.78)		Low (1.29,1.55)	Mid (1.55,1.65)	High (1.65,1.87)	
*n*	527	526	527		527	526	527		527	526	527		529	525	526	
Mean age (SD)	75.4 (6.8)	74.5 (6.0)	73.5 (5.3)	0.46	73.3 (5.7)	74.2 (5.9)	75.8 (6.4)	<0.01	74.1 (6.4)	74.8 (6.1)	74.5 (5.8)	0.164	75.0 (6.1)	74.6 (6.4)	73.8 (5.8)	<0.01
Females (%)	233 (44.2)	255 (48.5)	340 (64.5)	<0.01	437 (82.9)	253 (48.1)	138 (26.2)	<0.01	298 (56.5)	291 (55.3)	239 (45.4)	<0.01	493 (93.2)	297 (56.6)	38 (7.2)	<0.01
Arab ethnicity (%)	52 (9.9)	82 (15.6)	95 (18.0)	<0.01	37 (7.0)	70 (13.3)	122 (23.1)	<0.01	29 (5.5)	75 (14.3)	125 (23.7)	<0.01	61 (11.5)	75 (14.3)	93 (17.7)	0.02
Smoking (%)				0.09				<0.01				<0.01				<0.01
Current	71 (13.7)	56 (10.7)	45 (8.6)		50 (9.5)	56 (10.7)	66 (12.5)		54 (10.3)	57 (10.9)	61 (11.6)		38 (7.2)	65 (12.4)	69 (13.1)	
Past	182 (34.6)	194 (37.0)	181 (34.4)		135 (25.7)	172 (32.8)	250 (47.5)		159 (30.3)	182 (34.7)	216 (41.1)		107 (20.3)	174 (33.2)	276 (52.6)	
Never	273 (51.9)	274 (52.3)	300 (57.0)		341 (64.8)	296 (56.5)	210 (39.9)		312 (59.4)	286 (54.5)	249 (47.3)		382 (72.5)	285 (54.4)	180 (34.3)	
Mean number of comorbidities (SD)	2.8 (1.7)	3.1 (1.8)	3.4 (1.9)	<0.01	3.1 (1.7)	3.2 (1.8)	3.1 (2.0)	0.37	2.9 (1.7)	3.2 (1.8)	3.3 (2.0)	<0.01	3.4 (1.9)	3.0 (1.8)	2.9 (1.8)	<0.01
Mean MMSE (SD)	27.7 (2.7)	27.6 (2.9)	27.2 (3.2)	0.02	27.6 (2.7)	27.8 (2.8)	27.1 (3.3)	<0.01	27.9 (2.5)	27.7 (2.7)	26.9 (3.5)	<0.01	26.6 (3.4)	27.5 (3.0)	28.4 (2.0)	<0.01
SES (%)				<0.01				<0.01				<0.01				<0.01
Low	147 (27.9)	190 (36.1)	217 (41.2)		148 (28.1)	178 (33.8)	228 (43.3)		126 (23.9)	181 (3.4)	247 (46.9)		201 (38.0)	184 (35.0)	169 (32.1)	
Mid	214 (40.6)	182 (34.6)	203 (38.5)		208 (39.5)	198 (37.6)	193 (36.6)		211 (40.0)	197 (37.5)	191 (36.2)		216 (40.8)	191 (36.4)	192 (36.5)	
High	166 (31.5)	154 (29.3)	107 (20.3)		187 (35.5)	150 (28.5)	106 (20.1)		190 (36.1)	148 (28.1)	89 (16.9)		112 (21.2)	150 (28.6)	165 (31.4)	

*Not adjusted for age and sex. ABSI, a body shape index; ARI, anthropometric risk index; BMI, body mass index; MMSE, mini-mental state examination; SES, socioeconomic status; SD, standard deviation.

Correlation coefficients between the anthropometric measurements were assessed (Table 2). There was a weak correlation between BMI and ABSI Z scores (*r* = −0.11) and a strong correlation between BMI and WC (*r* = 0.71).

**Table 2:** Correlation matrix between anthropometric measures.

Pearson’s	BMI	ARI	ABSI	Height	WC	Weight
BMI	1	0.47	−0.17	−0.15	0.69	0.77
ARI	0.48	1	0.54	0.02	0.72	0.42
ABSI	−0.11	0.58	1	0.29	0.55	0.05
Height	−0.04	−0.06	−0.02	1	0.29	0.50
WC	0.71	0.72	0.55	0.10	1	0.79
Weight	0.78	0.42	0.01	0.38	0.79	1

Correlations (below-diagonal entries in table are correlations for z scores); ABSI, a body shape index; ARI, anthropometric risk index; BMI, body shape index; WC, waist circumference.

### Association between anthropometric indices and mortality

After a median follow-up of 13.2 (IQR 13.0;13.5) years, there were 757 deaths. Increased HRs for mortality were found for both ABSI and ARI in all models (Table 3). The minimally-adjusted association between BMI and mortality was attenuated in the fully adjusted model. Height was not associated with elevated risk in any of the models (Table 3). An analysis that further adjusted for smoking status and MMSE score resulted in similar results (Table S1).

**Table 3:** Hazard ratios for all-cause mortality associated with anthropometric indices.

	BMI	ABSI	ARI	Height
Model 1	1.12 (1.03;1.21)	1.16 (1.09;1.24)	1.27 (1.18;1.36)	0.97 (0.90;1.05)
Model 2	1.11 (1.02;1.20)	1.15 (1.07;1.23)	1.26 (1.17;1.35)	0.98 (0.90;1.06)
Model 3	1.07 (0.99;1.17)	1.13 (1.05;1.21)	1.23 (1.14;1.32)	0.98 (0.90;1.06)

Hazard ratios based on z scores; Model 1, age, sex, and ethnicity adjusted; Model 2, further adjusted for neighborhood SES score; Model 3, further adjusted for number of comorbidities. ABSI, a body shape index; ARI, anthropometric risk index; BMI, body mass index.

**Figure 2:**
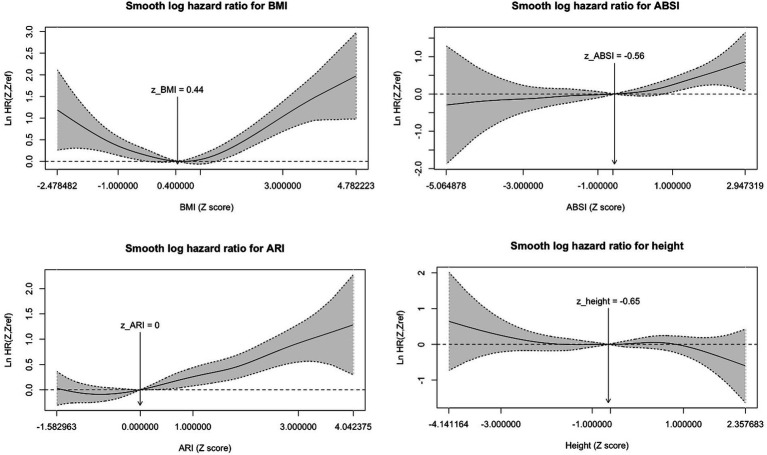
Adjusted smooth HR graphs for mortality. HR plotted against Z scores; Reference line set to mean Z score; ABSI, a body shape index; ARI, anthropometric risk index; BMI, body shape index; adjusted for age, sex, ethnicity, smoking status, SES, and comorbidities.

The log of the mortality HR adjusted for sociodemographic and clinical factors (model 3) was plotted against the Z score of each anthropometric measure (Figure 2). The graphs demonstrated a non-linear association for BMI, with a U-shaped relationship where the highest risk for mortality is for low and high values, and the lowest around the mean Z score value. In contrast, ABSI and ARI demonstrated a linear association, with a monotonic increase in the logarithmic risk of mortality with higher Z score values. Height had no significant association with mortality.

### Association between anthropometric indices and frailty

For the frailty risk analysis, we used all the participants who had Fried’s Biological Phenotype frailty score at T2, resulting in 554 participants, of whom 477 (86.1%) were categorized as non-frail and 77 (13.9%) as frail. Anthropometric indices assessed in T1 values were considered as predictors. We used IPW to minimize the selection bias due to attrition (Table 4). There was no significant association between BMI and frailty risk. In contrast, both ABSI and ARI were significantly associated with elevated frailty risk. Greater height was associated with a lower frailty risk. Further adjustments for SES score, smoking status, MMSE score, and comorbidities did not change the results materially. Additionally, there was no significant non-linear association between BMI and frailty in a spline model. The results of the unweighted analysis were similar, but with a slightly stronger association for ARI, and no association between height and frailty risk (Table S2).

**Table 4:** Odds ratios for frailty associated with anthropometric indices.

	BMI	ABSI	ARI	Height
Model 1	0.87 (0.73;1.04)	1.57 (1.37;1.81)	1.43 (1.22;1.68)	0.79 (0.67;0.92)
Model 2	0.90 (0.75;1.08)	1.60 (1.38;1.84)	1.49 (1.26;1.76)	0.80 (0.68;0.95)
Model 3	0.83 (0.69;1.01)	1.55 (1.34;1.79)	1.43 (1.21;1.70)	0.82 (0.70;0.97)

Odds ratios based on z scores; Model 1, age, sex and ethnicity adjusted; Model 2, further adjusted for SES neighborhood score and smoking status; Model 3, further adjusted for number of comorbidities and MMSE score. Propensity score and IPW were based on the Z scores of the anthropometrics measurements, age, sex, ethnicity, SES tertiles, smoking status, number of comorbidities, and MMSE score. IPW were calculated as $\left(\frac{\mathrm{follow}\;\mathrm{up}}{\mathrm{propensity}}\right)+\left(\frac{1-\mathrm{follow}\;\mathrm{up}}{1-\mathrm{propensity}}\right)$ . ABSI, a body shape index; ARI, anthropometric risk index; BMI, body mass index; MMSE, mini-mental state exam.

## Discussion

Mabat Zahav is an observational study of healthy aging in Israel, starting at the usual age of retirement and followed to the age approximating life expectancy. The current analysis was meant to determine the extent to which the basic anthropometric measures BMI and ABSI were predictive of longevity and of the absence of frailty. The correlation between BMI and WC was ~0.7, whereas the correlation between BMI and ABSI notably reduced to ~ − 0.1. In addition, ABSI, as in general populations, was predictive of mortality (8, 9, 24, 25). These results constitute the background for our expectation that ABSI could serve as a predictor for frailty, even after adjustment for some potential confounders. After multivariable adjustment, ABSI but not BMI was a predictor of frailty risk. This can be placed in the context of previous studies of the role of BMI and WC as risk factors for frailty. In a cross-section of Mabat Zahav (*n* = 1,619), BMI <30 and elevated WC were found to increase the incidence of frailty-associated disability (26, 27). However, the high mutual correlation between BMI and WC makes it difficult to determine the separate contributions of general and abdominal obesity (28). In the earlier cross-sectional English Longitudinal Study of Ageing (*n* = 3,055), BMI showed a U-shaped relationship to frailty, with the lowest risk BMI at 25–29.9. For abdominal obesity defined by WC thresholds, the risk for frailty was higher (29).

Using the Rockwood rather than Fried index, higher BMI was progressively associated with frailty in the Beijing Longitudinal Study on Aging II Cohort (*n* = 6,320). The relationship was similar but stronger for WC (30). A third cross-sectional survey, the Portuguese Nutrition UP 65 study (*n* = 1,444), found increased frailty was most prevalent when both BMI and WC were elevated (31).

Longitudinal studies of mortality and BMI and WC have been reviewed (32, 33). High mortality and disability have been documented prospectively for individuals with frailty (34). Longitudinal studies have further examined the relationship of anthropometrics to mortality in frail elderly, such as in the Women’s Health Initiative (*N* = 11,070), with higher BMI and lower waist-hip ratio being protective (35).

In a 5 year prospective Japanese study, dentition (*N* = 322) was identified as an important predictor of frailty (36). Observational data from India’s first nationally representative longitudinal aging survey found that elevated WC was associated with a lesser likelihood of successful aging (37).

Loss of lean tissue is the body composition hallmark of sarcopenia and is both a cause and marker of frailty (38, 39). While a marker of abdominal obesity, ABSI is sensitive to sarcopenia as it reflects fat-free mass as measured by bioelectrical impedance analysis (40). Mortality was doubled in men and increased 50% in women in a study from NHANES when sarcopenia defined by low limb lean tissue mass on dual-energy X-ray absorptiometry. DXA total body composition scan was accompanied by an elevated ABSI (41). Again, based on data from NHANES, body composition (*N* = 14,064) defined by DXA total body scan was found to be associated with allometric anthropometrics. Joint consideration of body composition with BMI and ABSI was found to improve mortality prediction (15, 24, 28, 42).

Inflammation has been identified as a risk factor for frailty (43). ABSI shows a positive association with C-reactive protein, a principal inflammation marker, suggesting another pathway that ABSI may mediate frailty (40). In the much larger context of the UK Biobank, ABSI was confirmed as a predictor of multiple inflammatory biomarkers (44). In a Korean series of patients having health checkups (*N* = 3,219), ABSI was predictive of MRI findings for cerebral small vessel disease including increased white matter intensity, silent infarctions, and cerebral microbleeds (45). In analysis of NHANES, abdominal aortic calcification estimated from lumbar spine DXA scans was strongly associated with above the mean ABSI (46). The “inflammatory” habit of smoking was also found to be associated with elevated ABSI in a recent population survey of middle aged men (47). Associations of ABSI along with BMI with diet composition have recently been reported (48). In terms of prevention, there is already evidence from Mabat Zahav for the association of both diet (19, 20) and physical activity (49) as modifiable mediators of healthy aging.

Notably, ARI, a hazard index based on both BMI and ABSI values, appeared to outperform ABSI as a mortality predictor (Table 3), but not as a frailty predictor (Table 4). This is consistent with the formulation of ARI used here having been derived specifically from mortality outcome data (18). A different, tailored function of ABSI and BMI attributable risk might improve frailty prediction further. Also, height was inversely associated with frailty in our weighted analysis. Previously, tall stature in CVD patients was associated with decreased risk of frailty in late life (50). While nutritional, metabolic, hormonal, socioeconomic, and genetic factors may be involved in this relationship, further support of this finding in future studies is warranted.

Strengths of this study include the longitudinal design and pre-planned evaluation of frailty according to the generally accepted Fried criteria. Weaknesses include that not all survivors (72.7%, see Figure 1) were assessed for frailty, despite the best efforts of the field investigators. However, similar loss at follow-up for the elderly has been reported for other recent studies (51). We attempted to address this methodological challenge by applying IPW. In addition, the small number of frailty subject at T2 (14%), while similar in previous analyses of the elderly population (3, 52), may have reflected on the statistical analysis. Unfortunately, hip circumference, which, like WC, can be normalized to BMI to give an additional central body index, was not measured in the current study (18, 53).

In this study comparing ABSI to BMI for mortality and frailty prediction in older adults, our findings hold clinical significance. ABSI demonstrates an independent association with mortality, suggesting its potential as a valuable supplement to BMI in evaluating health status among older individuals. Particularly noteworthy is ABSI’s superior predictive ability for frailty compared to BMI. These results underscore the need for a nuanced clinical approach, acknowledging the limitations of BMI and recognizing the distinctive insights offered by ABSI, especially in frailty risk assessment. Integrating ABSI measurements into risk stratification for older adults can enhance assessment precision, informing targeted interventions. Additionally, incorporating ABSI into public health screening initiatives may aid in identifying individuals at heightened risk, facilitating tailored preventive measures. While our study emphasizes the clinical and public health impact of considering ABSI alongside BMI, further investigation is required to explore its associations with other outcomes, such as cardiometabolic diseases, ensuring comprehensive preventive care for high-risk individuals.

## Conclusion

In a longitudinal study of a population cohort entering geriatric age, ABSI and BMI showed hazard ratios for mortality similar to those in general populations.

The odds ratio for frailty was not significant for BMI but was robust for ABSI and not reduced after adjustment for multiple confounders. In the quest for healthy aging, further study of ABSI as a predictor of frailty and other outcomes would seem warranted.

## Data availability statement

Publicly available datasets were analyzed in this study. This data can be found here: https://www.health.gov.il/UnitsOffice/ICDC/mabat/Pages/Mabat_Gold.aspx.

## Ethics statement

The studies involving humans were approved by Tel Aviv University Ethical Committee, Israel. The studies were conducted in accordance with the local legislation and institutional requirements. The participants provided their written informed consent to participate in this study.

## Author contributions

IS: Writing – original draft, Writing – review & editing. NK: Writing – review & editing. JK: Writing – original draft, Writing – review & editing. AG: Writing – review & editing. YG: Writing – original draft, Writing – review & editing.
