# Supplementary material for: The predictive ability of ABSI compared to BMI for mortality and frailty among older adults
Source: Front Nutr. 2024 Apr 12;11:1305330. doi: 10.3389/fnut.2024.1305330 (PMC11048479; doi:10.3389/fnut.2024.1305330)
Supplement: Supplementary file 1 [file Table_1.docx]

**Supplementary Table 1**

**Table S1. Hazard ratios for all-cause mortality associated with anthropometric indices**

|  | **BMI** | **ABSI** | **ARI** | **Height** |
| --- | --- | --- | --- | --- |
| **Model 1** | 1.11 (1.02;1.21) | 1.16 (1.09;1.24) | 1.27 (1.18;1.36) | 0.97 (0.90;1.05) |
| **Model 2** | 1.11 (1.03;1.21) | 1.14 (1.06;1.22) | 1.24 (1.16;1.34) | 0.98 (0.90;1.06) |
| **Model 3** | 1.07 (0.98;1.17) | 1.10 (1.03;1.18) | 1.20 (1.12;1.30) | 1.00 (0.92;1.08) |

*Hazard ratios based on Z scores****; Model 1****: age, sex and ethnicity adjusted;* ***Model 2****: further adjusted for SES neighborhood score and smoking status;* ***Model 3****: further adjusted for number of comorbidities and MMSE score. ABSI, a body shape index; ARI, anthropometric risk index; BMI, body mass index.*
