# Supplementary material for: The predictive ability of ABSI compared to BMI for mortality and frailty among older adults
Source: Front Nutr. 2024 Apr 12;11:1305330. doi: 10.3389/fnut.2024.1305330 (PMC11048479; doi:10.3389/fnut.2024.1305330)
Supplement: Supplementary file 2 [file Table_2.docx]

**Supplementary Table 2**

**Table S2. Odds ratios for frailty associated with anthropometric indices**

|  | **BMI** | **ABSI** | **ARI** | **Height** |
| --- | --- | --- | --- | --- |
| **Model 1** | 0.99 (0.73-1.34) | 1.56 (1.22-2.01) | 1.54 (1.16-2.03) | 0.93 (0.71-1.21) |
| **Model 2** | 0.98 (0.72-1.34) | 1.54 (1.19-1.98) | 1.56 (1.18-2.08) | 0.96 (0.73-1.25) |
| **Model 3** | 0.91 (0.66-1.25) | 1.59 (1.22-2.07) | 1.52 (1.13-2.05) | 0.97 (0.74-1.28) |
| *Odds ration based on Z scores;* ***Model 1****: age, sex, and ethnicity adjusted;* ***Model 2****: further adjusted for SES neighborhood score and smoking status;* ***Model 3****: further adjusted for number of comorbidities and MMSE score. *ABSI, a body shape index; ARI, anthropometric risk index; BMI, body shape index; MMSE, mini mental state exam.* | | | | |
